# Chemoprotective effect of nimbolide against N-methyl-N-nitrosourea induced gastric cancer via alteration of apoptosis and NF-κB signaling pathway

**DOI:** 10.1590/acb402125

**Published:** 2025-03-31

**Authors:** Yizhong Gu, Binguo Liu, Xiaoting Xia, Chunlei Luo, Yi Ren

**Affiliations:** 1 Shanghai No. 3 Rehabilitation Hospital – Department of Pain Rehabilitation – Shanghai – China.; 2 No. 983 Hospital of the Chinese People’s Liberation Army – Department of Pharmacy – Tianjin,300142 – China.; 3 Shanghai Integrated Traditional Chinese and Western Medicine Hospital – Department of Oncology – Shanghai – China.; 4 Fudan University – Jing’an District Central Hospital Affiliated – Department of Traditional Chinese Medicine – Shanghai – China.; 5 Shanghai Putuo District Hospital of Traditional Chinese Medicine – Department of Medical Ward – Shanghai – China.

**Keywords:** Stomach Neoplasms, Antioxidants, Inflammation, Apoptosis

## Abstract

**Purpose:**

Gastric cancer (GC) ranks as the third most common cause of cancer related mortality and as the fifth most frequently diagnosed cancer globally. Less than 30% of people with GC survive for more than five years.

**Methods:**

Nimbolide has been shown to have anticancer, anti-inflammatory, antiparasitic, and antioxidant properties. The current investigation showed the anticancer effect of nimbolide against N-methyl-N-nitrosourea (MNU) induced GC in rats. Rats were given MNU (100 mg/kg) orally to induce GC and received the oral administration of nimbolide (10, 20 and 40 mg/kg). The different biochemical parameters were estimated.

**Results:**

Nimbolide significantly (*p* < 0.001) altered the level of lactate dehydrogenase (LDH), alanine aminotransferase (ALT), aspartate aminotransferase (AST), alkaline phosphatase (ALP), cytochrome P450, cytochrome B5 and histone deacetylase (HDAC) activity. Nimbolide treatment significantly (p < 0.001) altered the level of antioxidant parameters like superoxide dismutase (SOD), glutathione peroxidase (GPx), catalase (CAT), malondialdehyde (MDA); cytokines such as tumor necrosis factor (TNF)-α, interleukin (IL)-1β, IL-2, IL-6; inflammatory parameters viz., cyclooxygenase-2 (COX-2), prostaglandin E2 (PGE2), vascular endothelial growth factor (VEGF), nuclear factor kappa-light-chain-enhancer of activated B cells (NF-κB) in the serum and stomach tissue. Nimbolide considerably altered (*p* < 0.001) the level of apoptosis parameters (Bcl-2, Bax and caspase-3), and the mRNA expression of VCAM-1, ICAM-1, TNF-α, IL-1β, IL-6, MCP-1, TLR4 and NF-κB.

**Conclusion:**

Nimbolide treatment considerably altered the GC against MNU induced GC via alteration of apoptosis and NF-κB signaling pathway.

## Introduction

Cancer is a significant global health challenge and one of the leading causes of mortality. Approximately 7.9 million deaths (13% of all deaths) occurred due to cancer. Reports suggest that 12 million deaths will increase until 2030^1^.

Gastric cancer represents a significant global health concern. It ranks as the second most deadly and fourth most frequently diagnosed cancer worldwide^2^. Gastric cancer affects various regions and demographic groups differently, yet it remains a serious health issue due to its substantial burden of illness and mortality^3^. Early detection and intervention play pivotal roles in improving outcomes for patients with gastric cancer^4^.

While the incidence of gastric cancer has declined over the past decade, it continues to be a significant cause of death and disease globally. Gastric cancer is widespread, with notably high rates observed in East Asia, particularly in countries like Japan and China. It typically follows a multistage progression, marked by stepwise changes in the gastric mucosa1. Before the onset of gastric carcinogenesis, several precursor lesions may emerge, including atypical hyperplasia, atrophy, metaplasia in the intestine, and alterations in gastric acidity. These early changes set the stage for the development and progression of gastric cancer^3^^,^ 4.

Gastric cancer progresses due to persistent genetic alterations that disrupt normal cell division, growth, survival, and blood vessel formation. The stomach lining is continually exposed to harmful reactive oxygen species (ROS) in the stomach cavity, originating from factors such as smoking, dietary intake, and inflammation induced by *Helicobacter pylori* infection^5^. Apoptosis and cell proliferation must remain balanced to maintain the health of mucosal tissue. When apoptosis decreases and proliferation increases, the risk of cancer development is elevated^6^^,^ 7. Preventing, delaying or reversing any of the stages of carcinogenesis can help stop the growth of aggressive gastric cancer^2^.

Currently, most gastric cancer cases (approximately 90%) are diagnosed at an advanced stage, in which the chances of survival are below 30%^8^. Some reports suggest that the progression and incidence of gastric cancer are primarily associated with the Nrf2, TOR, Notch, Wnt, and Hedgehog signaling pathways. Currently, surgery and chemotherapy are the main treatments for gastric cancer. However, these treatments have side effects, as they can be injurious and may lead to the development of resistance. Therefore, there is a crucial need to identify more potent and safer treatments for gastric cancer^1^^,^ 8.

It is widely known that the progression of gastric cancer is associated with the growth of new blood vessels, increased cell division, and impaired cell death^1^. Some studies indicate that inflammation is a key factor in cancer development^2^^,^ 3. Inflammation serves as a vital component of the immune response, encompassing both innate and adaptive immunity. Its primary objective is to protect the human body from various harmful agents. Serving as the initial reaction of the immune system, inflammation engages a variety of immune cells, cytokines, and chemokines that collectively promote the inflammatory process^9^. Various factors, including growth factors, DNA-damaging agents, inflammation, and stroma activation, can alter cell division and contribute to the development of abnormal functions and structures within cells. This can ultimately lead to cancer growth.

Inflammation and oxidative stress are closely associated with poorer outcomes in individuals with advanced stomach cancer^10^^,^ 11. Chemoprevention involves the utilization of specific natural or synthetic substances that can impede, decelerate, diminish, or prevent the progression of cancer from its precancerous stages to becoming invasive. Consequently, there is considerable interest in investigating the chemopreventive properties of nutritional agents, phytochemicals, and medicinal plants that serve as dietary antioxidants and can induce cell death (apoptosis). For these substances to be employed in clinical practice, they must demonstrate efficacy and safety through rigorous preclinical and clinical trials^5^^,^ 9 ^–^ 11.

Nimbolide is a chemical compound classified as a limonoid, which is isolated from plants in the Meliaceae family^10^^,^ 11. Nimbolide has been isolated from the neem tree (*Azadirachta indica*), which is native to India and other parts of Asia. Nimbolide has demonstrated anticancer, anti-inflammatory, antiparasitic, and antioxidant properties in various studies^12^^–^ 14. Zhang et al.^15^ exhibited the anti-obesity effect of nimbolide via alteration of Nrf2/HO-1 pathway, Ma et al.^12^ the protective effect of nimbolide against gestational diabetes mellitus via alteration of oxidative stress, inflammation and gut microbiota, and Ahmad et al.^16^ the protective effect against oxidative and genetics damage. Nimbolide can inhibit the activity of various enzymes and proteins that play key roles in cancer progression^17^^,^ 18. It can induce cell cycle arrest, apoptosis, and autophagy in various types of cancer cells. As a promising natural product, it holds potential applications in cancer therapy and prevention^12^^,^ 14 ^,^ 19.

The current investigation exhibited the anticancer effect of nimbolide against N-methyl-N-nitrosourea (MNU) induced gastric cancer (GC) rats via apoptosis and NF-κB signaling pathway.

## Methods

### Experimental animals

Male Swiss albino Wistar rats, weighing 150 ± 20 g and aged 12–14 weeks old, were procured from the departmental animal facility and housed in plastic cages in the same facility. The rats were kept under standard laboratory conditions, including a temperature of 22 ± 5°C, a light/dark cycle of 12/12 hours, and a relative humidity of 40–60%. They were provided with free access to standard laboratory food and water throughout the study period. The entire animal experiment was conducted in accordance with institutional guidelines.

### Induction of gastric cancer

For the induction of GC, the rats received the oral administration of 100 mg/kg MNU (dissolved in water) via oral gavage at 10-day intervals^1^.

### Test drug

The test drug (nimbolide) was administered to the rats in the form of an oral suspension. Nimbolide was dissolved in a 2% solution of Tween 80 to prepare the oral suspension.

### Experimental design

After the induction of GC in the rats, they were divided into different groups, as detailed in Table 1. The body weight of all rats in each group was measured at regular intervals, every 10 days, up to 60 days.

**Table 1:** Experimental group.

S. No	Group	Dose
1	Normal control	Vehicle
2	MNU	Vehicle
3	MNU + Nimbolide	10 mg/kg
4	MNU + Nimbolide	20 mg/kg
5	MNU + Nimbolide	40 mg/kg

MNU: N-methyl-N-nitrosourea, purchased from the Sigma Aldrich (United States of America). Source: Elaborated by the authors.

### Serum preparation

The rats were anesthetized using diethyl ether, and blood samples were collected from the inferior vena cava. The serum was separated by centrifuging the blood samples at 5,000 g for 15 minutes at 4°C.

### Tissue homogenate

The rats were fasted overnight and euthanized via cervical dislocation following anesthesia with ketamine (35–50 mg/kg) and xylazine (5–10 mg/kg). Gastric tissue was extracted and rinsed with phosphate-buffered saline (PBS), after which it was homogenized. The tissue homogenate was then centrifuged at 10,000 rpm for 10 minutes and stored at -4°C.

### Histone deacetylase and lactate dehydrogenase activity

The activity of histone deacetylase (HDAC) was estimated using a colorimetric kit according to the manufacturer’s instructions (Upstate, CA, United States of America).

The activity of lactate dehydrogenase (LDH) in the rat serum was assessed using an LDH kit. The LDH activity was determined by measuring the absorbance at 340 nm using a ultraviolet (UV) spectrophotometer^20^.

### Oxidative stress parameters

The levels of oxidative stress parameters, including superoxide dismutase (SOD), glutathione peroxidase (GPx), malondialdehyde (MDA), and glutathione (GSH), were assessed using kits following the manufacturer’s instructions (Nanjing Jiancheng Bioengineering Institute, China).

### Detoxification enzymes

Phase I enzymes, including cytochrome b5 and cytochrome P450, were quantified using a previously reported method with minor modifications^21^.

### Hepatic parameters

The hepatic parameters such as aspartate aminotransferase (AST), alkaline phosphatase (ALP), and alanine transaminase (ALT), were estimated using the kits following the manufacturer’s instructions (Nanjing Jiancheng Bioengineering Institute, China).

### Apoptosis parameters

Apoptosis parameters like caspase-3, Bax and Bcl-2 were determined using the enzyme-linked immunosorbent assay (ELISA) kit (Nanjing Jiancheng Bioengineering Institute, Nanjing, China).

### Cytokines and inflammatory mediators

The cytokines such as tumor necrosis factor (TNF)-α, interleukin (IL)-1β, -2, -6 and inflammatory mediators viz., vascular endothelial growth factor (VEGF), cyclooxygenase-2 (COX-2), prostaglandin E2 (PGE2) and nuclear factor Kappa-light-chain-enhancer of activated B cells (NF-κB) were estimated using the ELISA kit (Nanjing Jiancheng Bioengineering Institute, China) via following the manufacture instruction.

### Reverse transcription polymerase chain reaction analysis

The TRIzol RNA extraction kit was utilized for isolating total RNA following the manufacturer’s instructions (Qiagen, Hilden, Germany). Subsequently, cDNA was synthesized from the extracted RNA using a commercially available polymerase chain reaction (PCR) kit according to the manufacturer’s instructions (Thermo-Fisher Scientific, Waltham, United States of America). The primers used are presented in Table 2, with β-actin serving as the internal standard. All experiments were conducted in triplicate.

**Table 2:** List of primers.

S. No	Gene	Primers	
		Sense	Antisense
1	VCAM-1	GGTATCCCATCACTTGAGCAGG	TGAACCCAAACAGAGGCAGAGT
2	ICAM-1	CAATTTCTCATGCCGCACAG	CAATTTCTCATGCCGCACAG
3	IL-1β	TTGTCGAGATGCTGCTGTGA	CAGCTTTCGACAGTGAGGAGA
4	TNF-α	GCTGGCACCACTAGTTGGTTGT	TGTAGCCCACGTCGTAGCAAA
5	MCP-1	CCACAATGGTCTTGAAGATCAC	CCCCAGTCACCTGCTGTTAT
6	TLR4	ATGAAGATGCCAGAGCGGCTA	AGTGTATCGGTGGTCAGTGTGCTT
7	IL-6	ACAGAGGGATATCTATCAGGG	GCGCAAAAGTGAGCTCCAGA
8	NF-κB	ATCCCATCTTTGACAATCGTGC	CTGGTCCCGTGAAATACACCTC
9	β-actin	GCTCCTCCTGAGCGCAAGT	TCGTCATACTCCTGCTTGCTGAT

IL: interleukin; TNF: tumor necrosis factor; NF-κB: nuclear factor Kappa-light-chain-enhancer of activated B cells. Source: Elaborated by the authors.

### Statistical analysis

Graph Pad Prism Software (verson 8, St. Louis, United States of America) was used in this experimental study, and the whole data were presented as mean ± standard deviation (SD). The results of the study were analysed using the one-way analysis of variance followed by Dunnett’s t test, *p*<0.05 was consider as significant.

## Results

### Body and tumor weight

**Figure 1:**
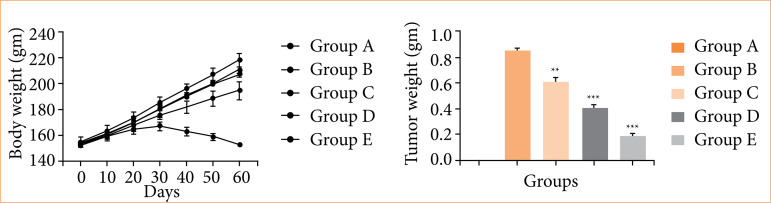
The body weight and tumor weight against N-methyl-N-nitrosourea (MNU) induced gastric cancer in rats. **(a)** Body weight and **(b)** tumor weight. Values are expressed as means ± standard error of the mean of six rats (n = 6) from each group.

Figure 1 demonstrates the reduction in body weight in group B (negative control), while nimbolide treatment significantly improves body weight.

*p < 0.05: significant; **p < 0.01: more significant; ***p < 0.001: extreme significant. Source: Elaborated by the authors.

Group A (normal control) rats did not show any tumor, group B (negative control) rats demonstrated the enhanced tumor weight (0.86 ± 0.004 g), and nimbolide-treated rats (groups C, D, and E) significantly (*p* < 0.001) reduced the tumor weight (0.61 ± 0.001 g, 0.41 ± 0.002 g, and 0.19 ± 0.003 g).

### Lactate dehydrogenase and histone deacetylase activity

**Figure 2:**
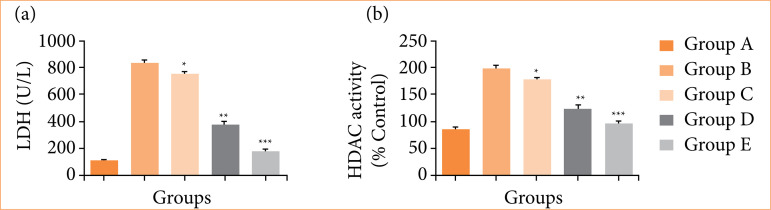
The lactate dehydrogenase (LDH) and histone deacetylase (HDAC) against N-methyl-N-nitrosourea (MNU) induced gastric cancer in rats. **(a)** LDH and **(b)** HDAC activity. Values are expressed as means ± standard error of the mean of six rats (n = 6) from each group.

Group B (negative control) rats exhibited the boosted level of LDH (Fig. 2a) and HDAC (Fig. 2b), and group C-E rats remarkably (*p* < 0.001) suppressed the level of LDH (187 ± 5.42 U/L) and HDAC activity (96.66 ± 2.56).

*p < 0.05: significant; **p < 0.01: more significant; ***p < 0.001: extreme significant. Source: Elaborated by the authors.

### Hepatic parameters

**Figure 3:**
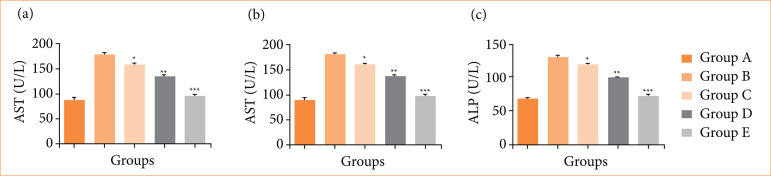
The hepatic parameters levels against N-methyl-N-nitrosourea (MNU) induced gastric cancer in rats. **(a)** aspartate aminotransferase (AST), **(b)** AST and **(c)** alkaline phosphatase (ALP). Values are expressed as means ± standard error of the mean of six rats (n = 6) from each group.

In comparing to group B (negative control), the levels of AST (Fig. 3a), ALT (Fig. 3b), and ALP (Fig. 3c) were significantly (*p* < 0.001) reduced by nimbolide treatment (10, 20, and 40 mg/kg).

*p < 0.05: significant; **p < 0.01: more significant; ***p < 0.001: extreme significant. Source: Elaborated by the authors.

### Phase I enzymes

**Figure 4:**
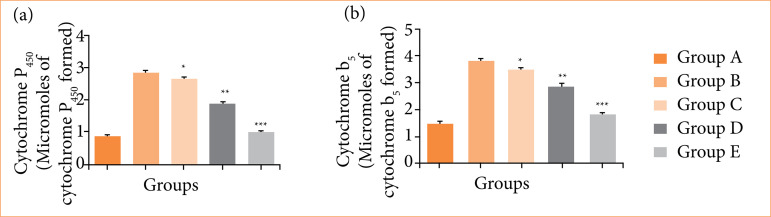
The phase I enzyme levels against N-methyl-N-nitrosourea (MNU) induced gastric cancer in rats. **(a)** cytochrome P450 and **(b)** cytochrome B5. Values are expressed as means ± standard error of the mean of six rats (n = 6) from each group.

The level of phase I enzymes like cytochrome P450 (Fig. 4a), cytochrome B5 (Fig. 4b) boosted in the group B (negative control) as compared to group A (normal control). Group C-E (nimbolide 10, 20 and 40 mg/kg) significantly (p < 0.001) suppressed the level of phase I enzymes. 

*p < 0.05: significant; **p < 0.01: more significant; ***p < 0.001: extreme significant. Source: Elaborated by the authors.

### Oxidative stress parameters

**Figure 5:**
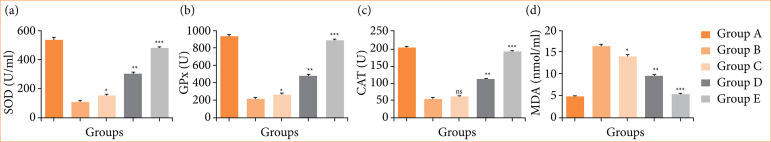
The antioxidant parameters levels in serum against N-methyl-N-nitrosourea (MNU) induced gastric cancer in rats. **(a)** Superoxide dismutase (SOD), **(b)** glutathione peroxidase (GPx), **(c)** catalase (CAT), and **(d)** malondialdehyde (MDA). Values are expressed as means ± standard error of the mean of six rats (n = 6) from each group.

Group B (negative control) rats exhibited the altered level of SOD (Fig. 5a), GPx (Fig. 5b), catalase (CAT) (Fig. 5c), MDA (Fig. 5d), and groups C–E (nimbolide 10, 20, and 40 mg/kg) significantly (*p* < 0.001) restored the levels of oxidative stress parameters in the serum.

*p < 0.05: significant; **p < 0.01: more significant; ***p < 0.001: extreme significant. Source: Elaborated by the authors.

**Figure 6:**
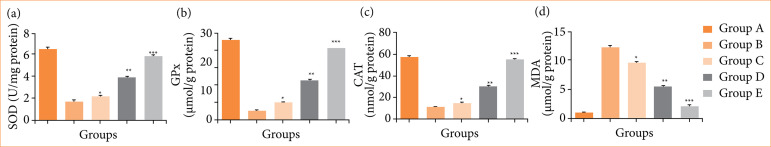
The antioxidant parameters level in stomach against N-methyl-N-nitrosourea (MNU) induced gastric cancer in rats. **(a)** Superoxide dismutase (SOD), **(b)** glutathione peroxidase (GPx), **(c)** catalase (CAT), and **(d)** malondialdehyde (MDA). Values are expressed as means ± standard error of the mean of six rats (n = 6) from each group.

Group B (negative control) rats remarkably modulated the level of SOD (Fig. 6a), GPx (Fig. 6b), CAT (Fig. 6c), MDA (Fig. 6d), and groups C–E (nimbolide 10, 20 and 40 mg/kg) significantly (*p* < 0.001) restored the level of oxidative stress parameters in the stomach tissue.

*p < 0.05: significant; **p < 0.01: more significant; ***p < 0.001: extreme significant. Source: Elaborated by the authors.

### Cytokines and inflammatory parameters

**Figure 7:**
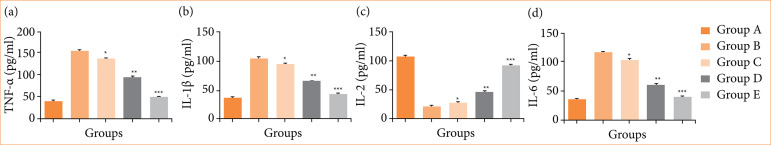
The cytokines level in serum against N-methyl-N-nitrosourea (MNU) induced gastric cancer in rats. **(a)** Tumor necrosis factor (TNF)-α, **(b)** interleukin (IL)-1β, **(c)** IL-2 and **(d)** IL-6. Values are expressed as means ± standard error of the mean of six rats (n = 6) from each group.

MNU group rats exhibited altered levels of TNF-α (Fig. 7a), IL-1β (Fig. 7b), IL-2 (Fig. 7c), and IL-6 (Fig. 7d) in the stomach as compared to group A (normal control), and nimbolide-treated rats significantly (*p* < 0.001) restored the levels of cytokines.

*p < 0.05: significant; **p < 0.01: more significant; ***p < 0.001: extreme significant. Source: Elaborated by the authors.

**Figure 8:**
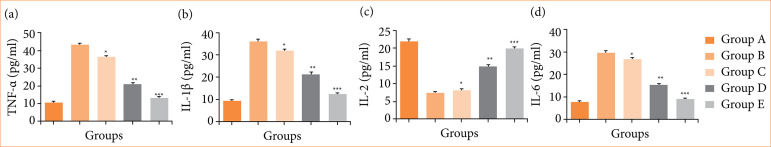
The cytokines level in stomach against N-methyl-N-nitrosourea (MNU) induced gastric cancer in rats. **(a)** Tumor necrosis factor (TNF)-α, **(b)** interleukin (IL)-1β, **(c)** IL-2 and **(d)** IL-6. Values are expressed as means ± standard error of the mean of six rats (n = 6) from each group.

MNU group rats exhibited the altered level of TNF-α (Fig. 8a), IL-1β (Fig. 8b), IL-2 (Fig. 8c), and IL-6 (Fig. 8d) in the stomach as compared to group A (normal control), and nimbolide treated group rats significantly (*p* < 0.001) restored the level of cytokines. 

*p < 0.05: significant; **p < 0.01: more significant; ***p < 0.001: extreme significant. Source: Elaborated by the authors.

**Figure 9:**
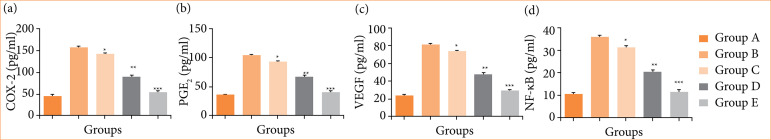
The inflammatory parameters level in serum against N-methyl-N-nitrosourea (MNU) induced gastric cancer in rats. **(a)** Cyclooxygenase-2 (COX-2), **(b)** prostaglandin E2 (PGE2), **(c)** vascular endothelial growth factor (VEGF), and **(d)** nuclear factor Kappa-light-chain-enhancer of activated B cells (NF-κB). Values are expressed as means ± standard error of the mean of six rats (n = 6) from each group.

Group B (negative control) rats exhibited the boosted level COX-2 (Fig. 9a), PGE2 (Fig. 9b), VEGF (Fig. 9c), and NF-κB (Fig. 9d), and groups C–E group rats significantly (*p* < 0.001) suppressed the level of inflammatory parameters in the serum.

*p < 0.05: significant; **p < 0.01: more significant; ***p < 0.001: extreme significant. Source: Elaborated by the authors.

**Figure 10:**
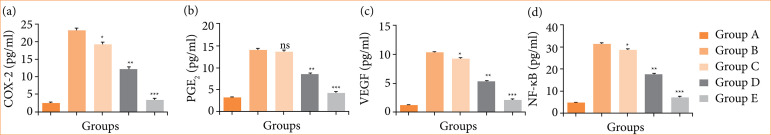
The inflammatory parameters level in stomach against N-methyl-N-nitrosourea (MNU) induced gastric cancer in rats. **(a)** Cyclooxygenase-2 (COX-2), **(b)** prostaglandin E2 (PGE2), **(c)** vascular endothelial growth factor (VEGF), and **(d)** nuclear factor Kappa-light-chain-enhancer of activated B cells (NF-κB). Values are expressed as means ± standard error of the mean of six rats (n = 6) from each group.

Group B (negative control) rats revealed elevated levels of COX-2 (Fig. 10a), PGE2 (Fig. 10b), VEGF (Fig. 10c), and NF-κB (Fig. 10d), and groups C–E rats significantly (*p* < 0.001) suppressed the levels of inflammatory parameters in the serum.

*p < 0.05: significant; **p < 0.01: more significant; ***p < 0.001: extreme significant. Source: Elaborated by the authors.

### Apoptosis

**Figure 11:**
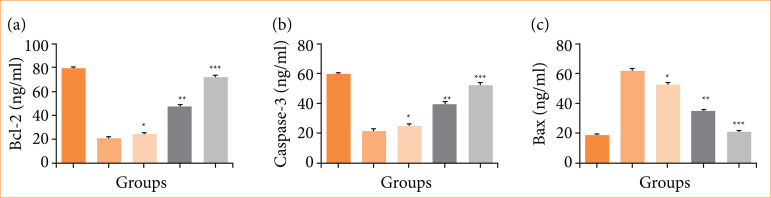
The apoptosis parameters level against N-methyl-N-nitrosourea (MNU) induced gastric cancer in rats. **(a)** Bcl-2, **(b)** caspase-3, and **(c)** Bax. Values are expressed as means ± standard error of the mean of six rats (n = 6) from each group.

Group B (negative control) rats presented the altered level of apoptosis parameters like Bcl-2 (Fig. 11a), caspase-3 (Fig. 11b), Bax (Fig. 11c) and nimbolide treatment significantly (*p* < 0.001) modulated the apoptosis level.

*p < 0.05: significant; **p < 0.01: more significant; ***p < 0.001: extreme significant. Source: Elaborated by the authors.

### mRNA expression

**Figure 12:**
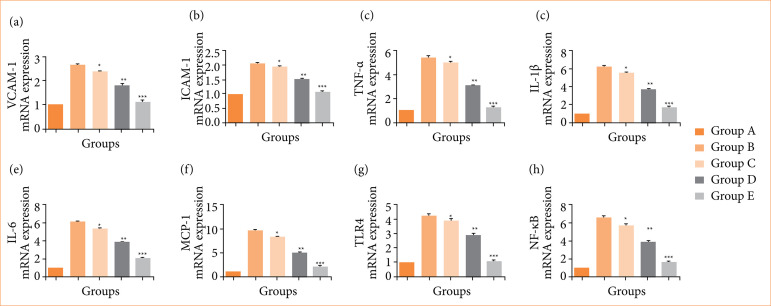
The mRNA expression level against N-methyl-N-nitrosourea (MNU) induced gastric cancer in rats. **(a)** VCAM-1, **(b)** ICAM-1, **(c)** tumor necrosis factor (TNF)-α, **(d)** interleukin (IL)-1β, **(e)** IL-6, **(f)** MCP-1, **(g)** TLR4 and **(h)** nuclear factor Kappa-light-chain-enhancer of activated B cells (NF-κB). Values are expressed as means ± standard error of the mean of six rats (n = 6) from each group.

Group B (negative control) rats demonstrated the increased mRNA expression of VCAM-1 (Fig. 12a), ICAM-1 (Fig. 12b), TNF-α (Fig. 12c), IL-1β (Fig. 12d), IL-6 (Fig. 12e), MCP-1 (Fig. 12f), TLR4 (Fig. 12g), and NF-κB (Fig. 12h), and nimbolide treatment significantly (*p* < 0.001) suppressed the mRNA expression. 

*p < 0.05: significant; **p < 0.01: more significant; ***p < 0.001: extreme significant. Source: Elaborated by the authors.

## Discussion

Previous reports highlighted that gastrointestinal cancer, notably GC, stands as one of the primary causes of death globally^1^. GC poses a significant challenge among all types of cancers, characterized by a decreased survival rate primarily attributed to late diagnosis and inherent resistance to existing chemotherapeutic drugs^8^. GC cases are predominantly adenocarcinomas, with a five-year survival rate typically ranging between 5 and 10%^21^. The current available treatments for GC include surgery and chemotherapy, yet both treatments have limitations due to their effects on non-cancerous cells^1^. Hence, there is an urgent need to explore potential treatment approaches for GC that can effectively halt its progression with minimal side effects. Many plant-derived drugs, numbering more than 100, are already utilized to treat various types of cancer. In this study, we aimed to investigate the anti-cancer effect of nimbolide on GC induced by MNU in rats.

Histone acetylation (HA) is an epigenetic modification that affects gene expression by altering the structure of chromatin, histone proteins, and DNA complexes^22^^,^ 23. HA plays a crucial role in the expansion and progression of various types of cancer by influencing the expression of tumor suppressor genes and oncogenes^24^. HA controls two types of enzymes: HDACs and histone acetyltransferases (HATs), which respectively add or remove acetyl groups from histones. Disruption of HA can lead to the activation of genes involved in cell division, growth, spreading, angiogenesis, and tissue invasion, or the inhibition of genes that regulate these processes25. The enzyme chymotrypsin catalyzes the cleavage of peptide bonds through hydrolysis, a process that occurs significantly faster in the presence of the enzyme compared to the uncatalyzed reaction1. During cancer, the level of HDAC increases, prompting researchers to target the HDAC receptor for cancer treatment and nimbolide treatment suppressed the activity of HDAC.

LDH is an enzyme that facilitates the interconversion of pyruvate and lactate. It comprises five isoforms, each with distinct functions and expression patterns. Elevated LDH levels in the blood are associated with worse outcomes in various types of cancer, including GC^1^. LDH is implicated in various processes that promote cancer, including altered energy metabolism in cancer cells, tumor cell invasion and metastasis, and suppression of the immune system^26^. Cytochrome P450 comprises a superfamily of enzymes responsible for metabolizing a wide range of endogenous and exogenous substances, including drugs. Certain cytochrome P450 enzymes have the ability to either activate or deactivate carcinogens, thereby influencing the risk and progression of gastric cancer^27^. CYP1A1, CYP1A2, and CYP1B1 are induced by polycyclic aromatic hydrocarbons, potent carcinogens capable of generating reactive intermediates that bind to DNA and induce mutations. Cytochrome b5, a hemoprotein, interacts with cytochrome P450, modulating its activity. Depending on the specific enzyme and substrate involved, cytochrome b5 can either enhance or inhibit the oxidation of substrates by cytochrome P450^27^^,^ 28.

Various epidemiological, pre-clinical and clinical reports suggest the relationship between the inflammation and cancer^29^^,^ 30. Inflammation can elevate the risk of cells becoming cancerous and developing into tumors. It serves as a significant factor in promoting cellular transformation into cancer cells and the formation of tumors. During inflammation, various cytokines and other inflammatory mediators affect multiple processes, including immune suppression, accelerated cancer cell proliferation, alterations in tissue structure, and the promotion of angiogenesis^31^^,^ 32. These factors collectively create a favorable environment for cancer growth and progression^9^.

During the early stages of cancer development, inflammation frequently serves as one of the initial triggers. This inflammatory response can establish a microenvironment conducive to tumor cell proliferation and survival. As inflammatory signals accumulate, they can stimulate the growth and expansion of cancerous cells, thereby laying the foundation for tumor development and progression. Cytokines such as TNF-α, IL-1β, IL-2, and IL-6 can enhance tumor growth, angiogenesis, metastasis, and invasion by promoting the survival and proliferation of tumor cells through activation of the NF-κB pathway^33^. They also induce the expression of metalloproteinases (MMP) and VEGF and suppress the anti-tumor immune response^34^^,^ 35. Hence, achieving a delicate balance between anti-inflammatory and pro-inflammatory cytokines within the tumor microenvironment is critical for determining the outcome of gastric cancer. Elevated levels of TNF-α, IL-1β, and IL-6 observed in the serum and stomach tissue are associated with advanced stages, poor survival outcomes, metastasis, lymph node involvement, and resistance to chemotherapy in patients with GC^1^.

Inflammation plays significant roles in the development and progression of gastric cancer, a type of cancer affecting the stomach. COX-2 is an enzyme that converts arachidonic acid into prostaglandins, such as PGE2. PGE2 is a lipid mediator that binds to specific receptors (EP1-4) and activates various signaling pathways. Overexpression of COX-2 and PGE2 is often observed in GC cells and tissues, in which they can promote tumor growth, invasion, angiogenesis, and metastasis. They achieve this by stimulating the proliferation and survival of tumor cells, activating the NF-κB pathway, inducing the expression of VEGF and MMPs, and suppressing the anti-tumor immune response^36^^,^ 37. Angiogenesis, the process of creating blood vessels, is driven by a growth factor called VEGF. Tumor cells heavily rely on angiogenesis for their survival and spread, as it provides them with oxygen and nutrients essential for growth, and facilitates their escape to other sites in the body^38^. NF-κB pathway plays a significant role in the development and progression of gastric cancer. NF-κB is a family of transcription factors that regulate various cellular processes, including inflammation, cell proliferation, apoptosis, and immune responses. Dysregulation of the NF-κB pathway has been implicated in the initiation, promotion, and metastasis of gastric cancer. Chronic inflammation is a major risk factor for GC development, and NF-κB is a key mediator of inflammatory responses.

Infection with *Helicobacter pylori*, a bacterium that colonizes the stomach and causes chronic gastritis, is strongly associated with GC. Activation of NF-κB by *H. pylori* infection promotes the production of pro-inflammatory cytokines. NF-κB regulates the expression of genes involved in cell proliferation and survival, such as cyclin D1, c-Myc, and anti-apoptotic proteins like Bcl-2 and Bcl-xL. Constitutive activation of NF-κB promotes uncontrolled cell proliferation and inhibits apoptosis, leading to tumor growth and progression. NF-κB regulates the expression of angiogenic factors such as VEGF and MMPs, which promote the formation of blood vessels to supply nutrients and oxygen to the growing tumor. Angiogenesis is crucial for tumor growth and metastasis, and NF-κB activation contributes to this process in GC. NF-κB is a transcription factor that governs the expression of genes implicated in a range of cellular processes, including inflammation, immunity, cell survival, and proliferation. It becomes activated in response to various stimuli such as cytokines, growth factors, and PGE2. In GC, NF-κB is often constitutively active in cancer cells and tissues, in which it regulates the expression of key molecules such as COX-2, PGE2, and VEGF. This activation contributes to the tumorigenic and metastatic potential of GC cells by promoting their survival, proliferation, invasion, angiogenesis, and evasion of the immune system^39^. GC rats treated with nimbolide remarkably suppressed the NF-κB signaling pathway.

Apoptosis, along with its associated alterations, plays a pivotal role in tumor progression and carcinogenesis. Two main pathways contribute to apoptosis: the intrinsic (or intracellular) pathway and the extrinsic (or death receptor) pathway. These pathways activate a cascade of caspases, which are proteases responsible for executing the apoptotic program by cleaving specific cellular substrates. Ultimately, both pathways converge on caspase activation, leading to programmed cell death^40^^,^ 41. Caspase 9 assumes a pivotal role as an initiator caspase. Its activation is triggered by the release of cytochrome-c from the mitochondria. Once activated, caspase 9 initiates the apoptosis of cells by activating effector caspases. In GC, alterations in the levels of apoptotic markers such as Bax, caspase 3, and caspase 9 are observed, with an imbalance typically favoring anti-apoptotic factors and suppressing pro-apoptotic signals. These changes contribute to the dysregulation of apoptosis and promote carcinogenesis^42^^–^ 44. Caspase 9 serves as a crucial initiator caspase in the intrinsic pathway of apoptosis. Its activation involves the formation of a complex called the apoptosome, consisting of apoptotic protease activating factor 1 (Apaf-1), dATP, and cytochrome c. This process is initiated by the release of cytochrome c from the mitochondria, facilitated by pro-apoptotic proteins like Bak and Bax, ultimately leading to the permeabilization of the mitochondrial membrane^42^^,^ 44. Report suggest that dysregulation of apoptosis during the GC condition occur due to imbalance between the anti and pro apoptotic factors9. The rats in the MNU group exhibited altered levels of apoptosis markers and showed activation of caspase 9. However, treatment with nimbolide significantly altered the levels of apoptosis markers, suggesting a protective effect against GC.

## Conclusion

Nimbolide remarkably boosted the body weight and decreased tumor weight. It also altered the levels of LDH, hepatic phase I enzymes, and HDAC activity. Additionally, nimbolide significantly affected oxidative stress parameters, cytokines, and inflammatory parameters in both serum and stomach tissue. Furthermore, it modulated apoptosis parameters along with suppressing mRNA expression of VCAM-1, ICAM-1, TNF-α, IL-1β, IL-6, MCP-1, TLR4, and NF-κB. In the future, we plan to investigate the chemoprotective effect of nimbolide via metabolomics.

## Data Availability

The data will be available on the request to the corresponding author.
